# A new method for continuous in vivo pH measurement in saliva and oral biofilm - a comparative pilot study

**DOI:** 10.1007/s00784-025-06703-9

**Published:** 2025-12-22

**Authors:** Antje Geiken, Ariel Salomon Gutman, Niklas Röder, Louise Holtmann, Christian Graetz, Karin Schwarz, Christof E. Dörfer

**Affiliations:** 1 Clinic of Conservative Dentistry and Periodontology, University of Kiel, Kiel, Germany; 2 Division of Food Technology, Institute of Human Nutrition and Food Science, Kiel University, Kiel, Germany

**Keywords:** PH value, Caries, Real-time monitoring, Nutrition, Erosion, Prevention

## Abstract

**Background:**

Measuring intraoral pH is a factor in determining the pathogenicity of processes. Until now, been continuous pH measurement over a period of 96 h in parallel (saliva [S]) and established biofilm [B]) has not been feasible. This exploratory study aimed to develop a method for continuous, wireless pH monitoring of both S and B.

**Methods:**

A wireless device with integrated pH probes was used to measure SpH and BpH in 12 participants (average age 24.28 ± 2.77 years) over a period of 96 h. Participants drank a glucose solution at three specified measurement points (G0: Baseline, G1: Glucose decline, G2: 30 min after drinking) and the 24-hour periods within the 96-hour measurement period were evaluated. The device was removed during meals and while brushing teeth.

**Results:**

Glucose intake significantly reduced pH in both (S + B) (*p* < 0.001). SpH was significantly higher (G0/ G1/ G2: 6.29 ± 0.29/ 5.55 ± 0.33/ 5.79 ± 0.32) than in BpH (G0/ G1/ G2: 6.03 ± 0.33/ 5.34 ± 0.41/ G2: 5.56 ± 0.32) at all three selected measurement points (p-value at G0/ G1/ G2: *p* = 0.003/ *p* = 0.005/ *p* = 0.002). Regardless of glucose intake, no statistical difference was found between SpH (0–24 h/ 24–48 h/ 48–72 h/ 72–96 h: 5.86 ± 0.41/ 5.75 ± 0.37/ 5.92 ± 0.38/ 5.90 ± 0.31), BpH (5.60 ± 0.52/ 5.59 ± 0.29/ 5.77 ± 0.39/ 5.70 ± 0.46) in the time periods (p-value 0–24 h/ 24–48 h/ 48–72 h/ 72–96 h: *p* = 0.09/ *p* = 0.27/ *p* = 0.40/ *p* = 0.27).

**Conclusions:**

The study design offers the possibility to continuously measure the pH value in S and B in the oral cavity over a period of 96 h.

**Clinical relevance:**

This wireless method developed for measuring pH in S and B can collect data under everyday conditions and has the potential to become a patient-friendly approach for pH monitoring in the future.

**Supplementary Information:**

The online version contains supplementary material available at 10.1007/s00784-025-06703-9.

## Background

Caries is a complex process that is influenced by the activity of the biofilm and the oral microbiome as well as the patient’s nutrition and social and economic conditions [1]. Carious processes are caused by a drop in pH due to metabolic products induced by bacteria of the oral biofilm. The critical intraoral pH for the development of dental caries is described as 5.5 for enamel [2], and 6.7 for radicular dentin [3]; below this thresholds, the hydroxyapatite begins to dissolve [4]. Due to its chemical, physical, and buffering properties, saliva is protective against the carious process under physiological conditions. In contrast, erosion on the tooth surface is caused directly by a low pH value in the oral cavity (e.g., orange juice) without any bacterial influence. This means that a reduction in the intraoral pH value caused by the action of acids of extrinsic origin (e.g., from the consumption of foods/juices) and/or of intrinsic origin (e.g., reflux) must be present for the development of erosion [5, 6]. Erosion is therefore a multifactorial process influenced by patient-related factors, such as salivary flow rate and buffer capacity, which affect its onset and progression [4].

Both caries and erosion negatively affect the tooth surface and can lead to substance defects. Low pH values can quickly lead to serious tooth defects, especially in younger children in the primary dentition, but also in older people due to exposed root surfaces [7, 8]. It is therefore desirable to be able to estimate how strong of a pH drop can be expected when consuming foods, as well as which foods can lead to a particularly strong pH drop due to bacterial metabolism. Products that do not further decrease the pH of 5.7 during consumption and for a period of 30 min after consumption have no significant cariogenic potential and are therefore considered “tooth-friendly” [9].

Methods for measuring the oral pH value were developed as early as 1965 [10]. Initially, glass electrodes were used, but due to their fragility, they posed a high risk of injury [11]. Currently, the effect of foods and medicines on saliva pH can be measured using intraoral pH telemetry. For this purpose, pH probes in intraoral devices measure the pH value in saliva during the consumption of the fermentable product [9]. However, this method is limited to patients wearing partial dentures and all measurements are done in a small number of such patients, who received a partial denture replacement with the probes embedded for measurements, and not representative for different age groups, especially younger patients. A disadvantage of this method is the necessary connection of the intraoral pH probe via a cable to a stationary receiver [12]. This means that data can only be obtained in a specific study design. Due to these limitations, it has not been possible to sufficiently measure pH value in an established biofilm. In particular, the possible interactions within the biofilm and their effects on the pH value have not yet been sufficiently investigated. Thus, a new wireless pH telemetry with a measurement duration of 96 h. is evaluated in this pilot study. To date, no scientific studies have been conducted using this method. For this purpose, two wireless pH probes were transferred to an intraoral device. Due to the delicate design of the appliance, which resembles orthodontic braces, partially edentulous patients were not required. In addition, these probes allowed continuous temporal measurement of pH over a 96-hour period and the growth of a biofilm relevant for a cariogenic process on the tip of the probe. The aim of this study was to test the methodology with regard to the validity and reliability of pH measurement in saliva and oral biofilm. The null hypothesis was that there are no differences between the pH values of saliva and biofilm.

## Materials and methods

The study was ethically approved by the University of Kiel (D 521/22) and conducted in accordance with the guidelines of the Declaration of Helsinki. The study was registered with the German Clinical Trial Registry (DRKS) and in the World Health Organization International Clinical Platform Search Portal (DRKS00030617) on 2 November 2022. All subjects were orally informed in detail about the objectives, implementation, and possible risks of the study and gave their written consent to participate. The study design was performed as a monocentric study in the context of quality assurance and reproducibility.

### Participants

The number of test individuals corresponded to previous studies with a similar research question [13, 14]. Individuals were included when presenting biofilm. Exclusion criteria for study participation were BMI < 18.5 or > 29.5 kg/m², metabolic or endocrine diseases, malabsorption syndrome, smoking, alcohol and drug abuse, medications influencing saliva production (e.g. antidepressants, antibiotics), presence of a defibrillator or pacemaker, nickel allergy, known allergies to the materials used, an MRI scan scheduled during the study period, reflux disease, bulimia, infectious medical conditions (e.g., HIV, hepatitis, or diabetes), and epilepsy. Further dental exclusion criteria were existing dental hard tissue defects requiring treatment, a periodontal screening index (PSI) > 2, professional dental cleaning less than four weeks prior, no stable occlusion, orthodontic treatment ongoing at the time of the study, wearing an occlusal splint, and an insufficient number of teeth (i.e., no terminal molar in the fourth or third quadrant or more than two missing teeth per quadrant). Dental professionals, including students, were also excluded. All subjects were informed in detail about the aims, conduct, and possible risks of the study during a personal interview and received a subject information sheet. Written consent to participate in the study was obtained from each subject.

### Intraoral appliance and pH telemetry

**Fig. 1:**
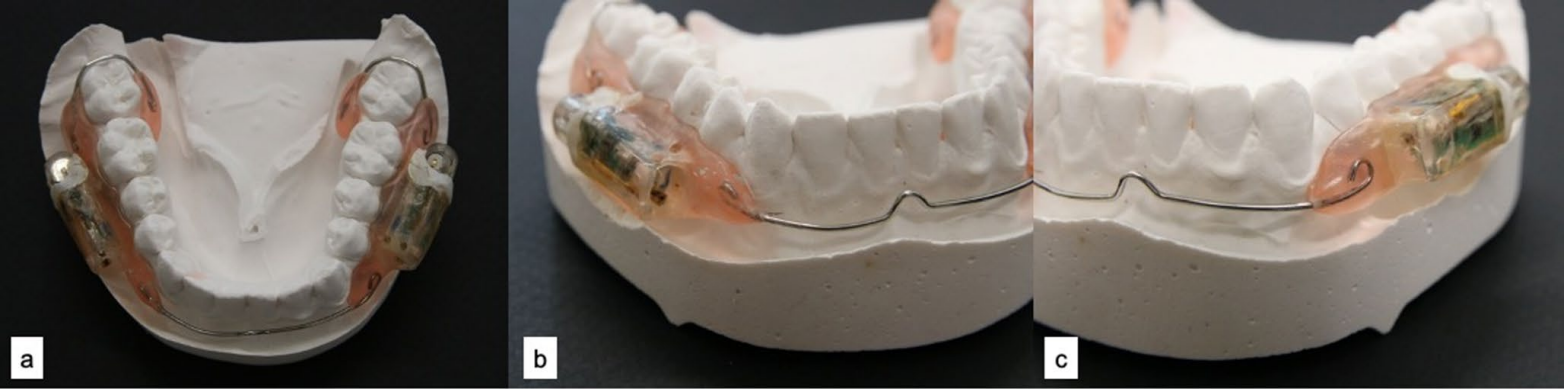
Intraoral device (after 14 days of wear) on a plaster model. (**a**) General view. (**b**) Probe for measuring the pH of saliva (**c**) Probe for measuring the pH of biofilm

**Fig. 2:**
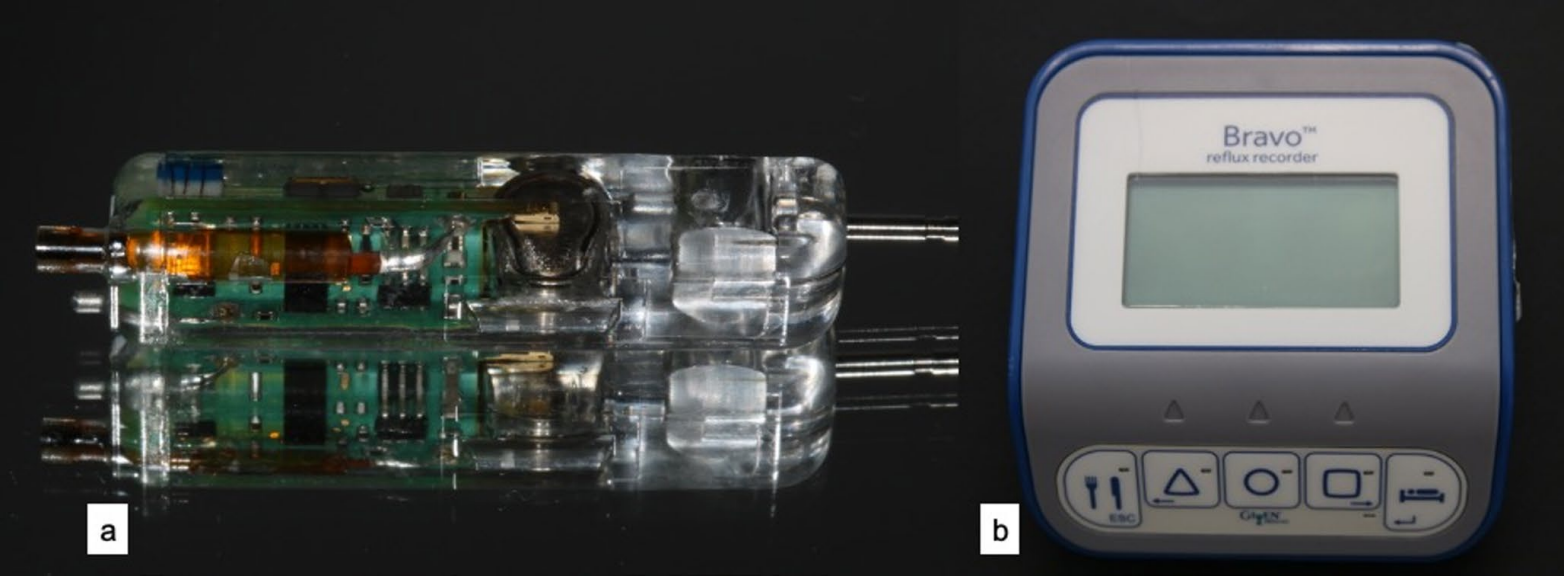
(**a**) pH probe (CE marking: G1 094769 0011 Rev. 00, Bravo, Medtronic Deutschland GmbH, Meerbusch) and (**b**) Data receiver (Bravo Reflux Recorder, Medtronic Deutschland GmbH, Meerbusch)

To fabricate the devices, impressions were taken of the subjects; upper and lower jaws (Xanatasil, Kulzer GmbH, Hanau), and plaster models were made. These devices were custom-made for each subject. Two quivers (size: 15 mm [length] x 8 mm [width] x 8 mm [height]) made of plastic (Dental LT, Clear Resin V2, form labs, Berlin, Germany) were attached to an apparatus worn in the lower jaw in the vestibular region 36 − 34 and 46 − 44. One quiver was perforated to ensure plaque accumulation and to measure the pH value in the biofilm later in the study. The second quiver for measuring the pH of saliva was designed to be open (Fig. 1). After a 10-day biofilm growth phase, the saliva quiver was cleaned, and the two quivers (saliva and biofilm) were each fitted with a battery-powered pH probe made of epoxy plastic (CE marking: G1 094769 0011 Rev. 00, Bravo, Medtronic Deutschland GmbH, Meerbusch; Fig. 2a) and placed over each quiver on the buccal surfaces of the appliance. The data were transmitted wirelessly to a data receiver (Bravo Reflux Recorder, Medtronic Deutschland GmbH, Meerbusch; Fig. 2b). The subjects were instructed to clean the outside of the quiver and the probe for measuring the saliva pH twice a day with water and a toothbrush. To do this, the device was removed from the mouth and cleaned extraorally by the test subjects until no deposits were visible to the naked eye. The device was then reinserted into the mouth. The test subjects were shown and instructed how to recognise the saliva quiver. The saliva quiver was open towards the oral cavity, while the biofilm quiver was closed (Figs. 1a, b). The subjects were informed of the importance of cleaning the saliva and were not to clean the biofilm pH probe.

### Validation of the pH sensor

To check the accuracy of the pH sensor, a validation of the pH probes was conducted before the study began. For this purpose, four probes were immersed in 2.00, 4.01, and 7.00 buffer solutions (Ph Buffer 4.01/ 7.00: Technical Buffer WTW, Xylem Analytics Germany GmbH, Weilheim, Germany, pH Buffer 2.00: Technical Buffer, Mettler Toledo GmbH, Greifensee, Switzerland) for five minutes each. After each measurement in the buffer solutions, the pH probe was neutralized for five minutes in a saline solution (0.9% Ecotainer, B. Braun Isotone Saline Solution, Melsungen, Germany).

A total of 28 measurement series were carried out. The standard deviation of the pH value across all buffer solutions was 0.14 from the actual value. The deviation at pH 4.01 was 4.22 (± 0.47); at pH 2, the actual pH value was 2.11 (± 0.14); and at pH 7, the actual pH value was 6.57 (± 0.14). Therefore, the actual and nominal values deviated only slightly and the probes were regarded as valid and reliable with respect to the study aims.

### Oral hygiene

During the entire trial period (14 days), subjects’ teeth were brushed with a predefined fluoride-containing standard toothpaste (Blend a med, Procter & Gamble, Schwalbach am Taunus, Germany) according to the normal oral hygiene recommendations (i.e., twice daily, once in the morning and once in the evening). The use of oral care products for the interdental space (interdental brushes, dental floss, or similar) was prohibited during the study period. The subjects were also asked not to have professional tooth cleaning or other dental treatments performed for four weeks before the start of the study and during the study.

### Trial procedure

As part of the preparatory measures, all pH probes used were tested for functionality before they were inserted into the test subjects’ rails. The subjects wore the appliance in the mandible for a total of 14 days. The first 10 days were used to ensure adequate plaque accumulation in the quivers. On day 10, the pH probes were inserted. One pH probe measured biofilm, while the other measured saliva. The subjects were not allowed to clean the devices; only the measuring unit of the pH probes, which measured saliva, was cleaned daily with a toothbrush and tap water. The appliance was worn continuously (96 h) by the subjects; it was removed only at mealtimes and during oral hygiene, during which time it was stored in 0.9% saline solution (Ecoflac Plus, B. Braun AG, Melsungen, Germany). The data collected during storage of the appliance in saline solution were not included in the evaluation.

During the four-day measuring phase, the subjects consumed 100 ml of glucose solution (Glucosteril 10%, Fresenius Kabi Deutschland GmbH, Bad Homburg, Germany) twice daily at standardized times (10:00 a.m. and 16:00 p.m.) and conditions (i.e., each sip was drunk without interruption and distributed in the mouth before swallowing). A waiting period of one hour was observed before consuming other food, beverages, or drinks, both before and after consuming the glucose solution. During the test phase, the subjects were not allowed to consume any other cariogenic beverages (juices, soft drinks, energy drinks, etc.). To record the timing of glucose solution and other beverage and food ingestion and to assess subjects’ compliance, participants kept a food diary throughout the study period. In addition, the subjects documented drinking the glucose solution by entering this into the pH probe receiver recording device. The pH values were measured in real time and could be read by the test subjects at any time on the recorder’s display. After four days of use, the appliance was removed. The data was downloaded from the recorder and a retrospective data evaluation was carried out.

Clinical data were also collected. A comprehensive dental examination was performed at each appointment at the beginning and at the end of the 14-days wearing time of the appliance, including an assessment of caries defects, the decayed, missing filled teeth (DMFT) Index, and an evaluation of mucosal lesions. Additional measurements comprised the Periodontal Screening Index (PSI), the plaque index (as described by Quigley and Hein and modified by Turesky), salivary flow rate and buffer capacity, and the gingivitis index, per to Löe and Silness [15, 16]. The plaque index was determined by staining the oral biofilm on six tooth surfaces (distobuccal, midbuccal, mesiobuccal, distolingual, midlingual, and mesiobuccal) with a staining solution (Mira-2-Ton, Hager Werken GmbH und Co. KG, Duisburg). The salivary flow rate was determined as stimulated salivation; the subjects chewed a paraffin block for one minute before the measurement and then spit it out for the one-minute saliva collection phase. To obtain unstimulated saliva, 5 ml was collected at rest (i.e., without active stimulation of saliva production). The buffering capacity of saliva was measured with unstimulated saliva using the method of Krasse [17] and modified according to Dodds, et al. [18].

### Data extraction and analysis

Due to the pilot character of the study, no sample size calculation was performed. Also, as not being an interventional study, per protocol analysis was performed. Data were initially transferred to the Reflux Software v6.1 (Medtronic GmbH, Meerbusch, Germany). Graphical representations were created using Microsoft Excel 2019 for Mac (Microsoft Corporation, Redmond, USA). The pH values determined were taken as primary outcome and statistically evaluated and presented side by side in a common figure for a period of 24 h or for the drinking period to allow better comparison between the groups (Figs. 1 and 2). Further statistical analyses were carried out in SPSS for Mac 28.0.0.0 (IBM, Chicago, IL, USA). The mean value and standard deviation were calculated for the dental parameters. A t-test, ANOVA, and Kruskal–Wallis test were used for the statistical analysis of group differences, depending on the data distribution. In addition, statistical analyses were performed in R (version 4.3.1) using the packages lme4 (version 1.1–37) and lmerTest (version 3.1-3) for fitting linear mixed effects and emmeans (version 1.11.2-8) for estimated marginal means and post hoc contrasts. The car package (version 3.1-3) was used for the variance analysis of the fixed effects. Linear mixed effects models were used to analyse the pH values at the different measurement points (baseline, decline and 30 min after glucose intake). The fixed effects included the biomatrix (saliva or biofilm) and the time point (morning, afternoon or evening). The subject ID was included as a random effect to account for inter-individual variation; α = 0.05 was defined as the significance level.

## Results

### Patient clinical data

**Fig. 3:**
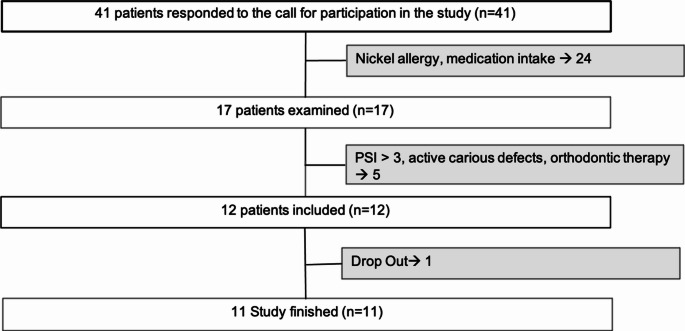
Flowchart of the subject search and selection results

A total of 12 generally healthy subjects (7 women and 5 men) without acute need for dental treatment and with an average age of 24.28 years (SD ± 2.77) were included in the study (Fig. 3; Table 1). One subject dropped out of the study early for personal reasons. A slight increase in the values of the clinical parameters (Plaque Index 1.62 ± 0.63/ 2.01 ± 0.63; Gingivitis Index 0.48 ± 0.33/ 1.25 ± 0.6) was observed. The values for the pH of saliva (7.56 ± 0.23; 5.44 ± 0.81/ 7.62 ± 0.32; 5.74 ± 0.53) and the rate of salivation (13.7 ± 6.76/ 14.4 ± 5.33) remained stable.

**Table 1:** Demographic and clinical data

SVariable and attribute	First Visit	Last Visit
Number of test subjects	12	11
Female	7	6
Male	5	5
Average age ± SD [range] (year)	24.28 ±2.77	-
	[21-31]	
DMFT^a^	6.5 ±6.3	-
	[0-13]	
Plaque index (Quigley and Hein)	1.62 ±0.63	2.01 ±0.63
	[0.11-2.57]	[0.42-3.44]
Gingivitis index (Löw and Silness)	0.48 ±0.33	1.25 ±0.6
	[0.08- 1.29]	[0.5-1.79]
Mean salivary flow (ml)	13.71±6.76	14.40±5.33
	[18.11-21.01]	[16.02-22.11]
Mean Saliva: pure and buffered pH	7.56±0.23	7.62 ±0.32
	[7.19-7.84]	[4.28-6.42]
	5.44 ±0.81	5.74 ±0.53
	[4.91-6.37]	[4.91-6.37]

mean (SD). The values in square brackets show the minimum and maximum values

^a^*DMFT* decayed missed filled teeth

### pH measurement biofilm and saliva

No statistical difference was found between the pH values of saliva and biofilm for the average minimum and maximum values between the time periods (0–24 h, 24–48 h, 48–72 h, 72–96 h) regardless of glucose intake (Tables 2, 3 and 4).

**Table 2:** pH values in saliva and biofilm at 24 h, 48 h, 72 h, and 96 h

Observation period (in h)	Saliva pH^a^			Biofilm pH^a^		
	Average	Minimum	Maximum	Average	Minimum	Maximum
0–24h	5.86 ± 0.41	4.46 ± 0.41	7.12 ± 0.52	5.60 ± 0.52	4.08 ± 0.53	7.32 ± 0.88
	[4.95–6.44]	[3.89–5.11]	[6.59–8.57]	[4.90–6.49]	[2.96–4.96]	[6.45–9.31]
24–48h	5.75 ± 0.37	4.44 ± 0.59	6.88 ± 0.26	5.59 ± 0.29	4.29 ± 0.38	6.85 ± 0.68
	[4.97–6.47]	[3.40–5.38]	[6.59–7.61]	[5.14–6.14]	[3.35–4.79]	[6.03–8.44]
48–72h	5.92 ± 0.38	4.66 ± 0.56	7.12 ± 0.52	5.77 ± 0.39	4.37 ± 0.49	6.93 ± 0.55
	[5.37–6.76]	[3.71–5.65]	[6.55–8.4]	[5.16–6.34]	[3.44–5.42]	[6.14–7.87]
72–96h	5.90 ± 0.31	4.73 ± 0.41	7.16 ± 0.43	5.70 ± 0.46	4.41 ± 0.46	6.94 ± 0.63
	[5.31–6.48]	[3.92–5.36]	[6.63–8.26]	[4.78–6.45]	[3.64–5.27]	[5.82–8.18]

^a^ mean (SD), The values in square brackets show the minimum and maximum values

The data collected during storage of the appliance in saline solution (food intake, oral hygiene) were not included in the evaluation

**Table 3:** Significance calculation pH values in saliva vs biofilm (average) during 24h–48, 48h–72h, and 72h–96h. Significant values are highlighted in bold

period of time (h)	Saliva vs Biofilm^b^ average		
	*t*-value	*F*-value	*p*-value
0–24	1.23	0.75	0.09
24–48	1.13	0.15	0.27
48–72	0.85	0.27	0.40
72–96	1.14	0.93	0.27

^b^t-test

The data collected during storage of the appliance in saline solution (food intake, oral hygiene) were not included in the evaluation

**Table 4:** Significance calculation of pH values in saliva and biofilm over the total period of 24 to 96 h. Significant values are highlighted in bold

period of time	Saliva^a^	Biofilm^a^
24h–96h	F-value	F-value
	p-value	p-value
average	*F* = 0.39	*F* = 0.42
	*p* = 0.76	*p* = 0.74
Minimal	*F* = 0.84	*F* = 1.00
	*p* = 0.48	*p* = 0.40
Maximal	*F* = 0.85	*F* = 0.90
	*p* = 0.48	*p* = 0.45

^a^Anova

The data collected during storage of the appliance in saline solution (food intake, oral hygiene) were not included in the evaluation

**Fig. 4:**
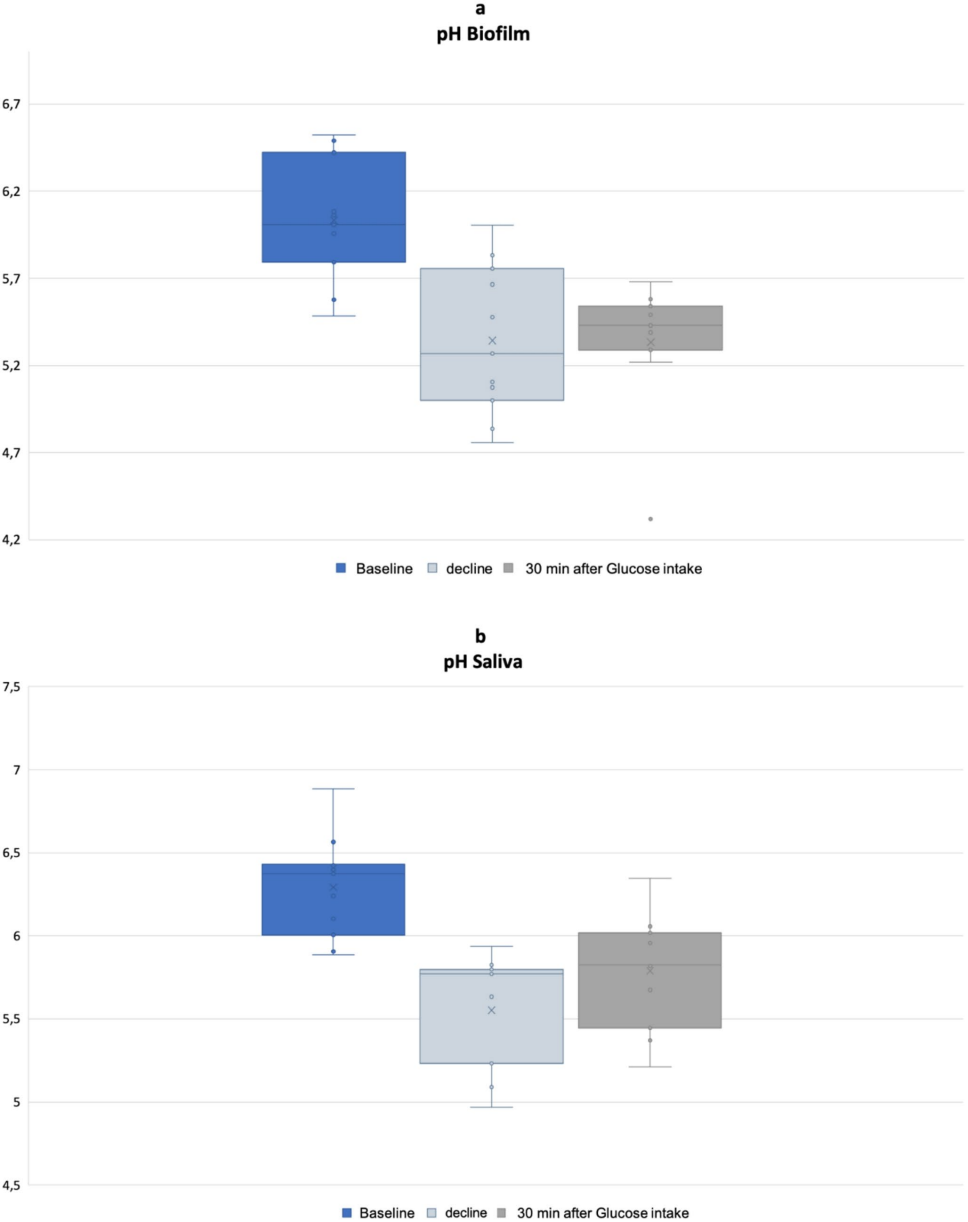
Reduction in pH value for both biomaterials (**a**) for biofilm and (**b**) for saliva from baseline to peak value. Baseline value Saliva/Biofilm (G0), glucose decline Biofilm/Saliva (G1), final pH (G2)

During glucose solution consumption, pH deviations in the biofilm and saliva were compared at different measurement points. A baseline pH value was obtained at the start of the drinking phase (lowest pH value +/- 1 min, baseline value – G0), the lowest pH value was measured within 30 min of consumption of the glucose drink (glucose decline – G1), and a final value was measured 30 min after consumption of the glucose solution (final pH – G2). The effect of glucose uptake on pH was compared for both biomaterials. In both cases, a significant reduction in pH was observed (*p* < 0.001; Fig. 4).

The pH value in saliva was significantly higher than in the biofilm at all three selected measuring points (p-value: G0 = 0.003, G1 = 0.005, G2 = 0.002; Table 5).

**Table 5:** pH values in saliva and biofilm during glucose consumption at the measurement points baseline

Measurement points	Saliva pH Mean ± SD [range]	Biofilm pH Mean ± SD [range]	Estimate (StErr)	DF-value	*t*-value^a^	*p*-value
Baseline (G0)	6.29 ± 0.29	6.03 ± 0.33	-0.24	96.05	-3.05	**0.003**
	[5.89–6.89]	[5.49–6.53]	(0.08)			
glucose decline (G1)	5.55 ± 0.33	5.34 ± 0.41	-0.18	95.94	-2.88	**0.005**
	[4.97–5.94]	[4.76–6.01]	(0.06)			
30 minutes after drinking (G2)	5.79 ± 0.32	5.56 ± 0.32	-0.20	94.53	-3.37	**0.002**
	[5.21–6.35]	[5.11–6.21]	(0.06)			

^a^paired t-Test

The data collected during storage of the appliance in saline solution (food intake, oral hygiene) were not included in the evaluation

Analysis of variance revealed a significant effect of biomatrix on pH values (Tables 6 and 7), whereas the time point (morning, afternoon or evening) had no significant effect. Estimated marginal means (emmeans) between saliva and biofilm samples showed that the biofilm had a consistently lower pH value across all measurements. At baseline, the average biofilm pH was 0.236 units lower than the average saliva pH. Similarly, the pH decline was 0.184 units lower in biofilm. 30 min after glucose consumption, the biofilm pH was still 0.19 units lower than the saliva pH.

**Table 6:** pH values in saliva and biofilm during glucose. Results of further analysis using a mixed model. Emmean, the model-based estimated marginal mean for the respective factor. This value corresponds to the expected average pH calculated from the model. Lower CL/ upper CL: the lower and upper limits of the 95% confidence interval around the estimated mean

Measurement points	Sample matrix	emmean	Lower CL	Upper CL
Baseline (G0)	biofilm	6.05	5.84	6.25
Baseline (G0)	saliva	6.28	6.08	6.49
glucose decline (G1)	biofilm	5.35	5.10	5.59
glucose decline (G1)	saliva	5.53	5.29	5.78
30 minutes after drinking (G2)	biofilm	5.58	5.34	5.81
30 minutes after drinking (G2)	saliva	5.77	5.53	6.00

**Table 7:** pH values in saliva and biofilm during glucose consumption at the measurement points baseline. Results of further analysis using a mixed model. Estimate: the estimated difference between the groups (biofilm and saliva). A negative value indicates that the value in the biofilm is lower than in saliva. Lower CL/ upper CL: the lower and upper limits of the 95% confidence interval around the estimated mean

Measurement points	contrast	estimate	SE	Lower CL	Upper CL	t ratio	p value
Baseline (G0)	biofilm-saliva	-0.236	0.077	-0.390	-0.083	-3.055	**0.003**
glucose decline (G1)	biofilm-saliva	-0.184	0.064	-0.312	-0.056	-2.858	**0.005**
30 minutes after drinking (G2)	biofilm-saliva	-0.190	0.068	-0.325	-0.055	-2.791	**0.006**

## Discussion

To the best of our knowledge, this study is the first to investigate a new method for measuring pH values in the oral cavity in biofilms and saliva using long-term measurements over 96 h in situ [19]. Previous studies to determine the pH value in the mouth—in both biofilm and saliva—have been on data transmission using a pH probe placed in the mouth and connected to a receiving device via a cable [9]. This approach, which does not allow for flexible data collection, is linked to a highly specific study setting, in which data collection must take place at a fixed location at the study site; thus, only a limited period of time can be covered [9]. A permanent measurement of pH value over the course of the day at several consecutive times, for instance, to distinguish between day and night rhythms, is not possible. In addition, due to the increased space requirements of the pH probe, a special patient clientele is required, namely patients with partial dentures. The patients must wear modified partial dentures in which the pH probes are embedded. Fully toothed, younger patients are therefore not suitable for this measurement method. In removable devices, similar to those in the current study design, the wearing time has so far been limited to a maximum of 19–24 h, which does not allow for constant monitoring or reflect the aspect of a growing biofilm [14, 20, 21]. The study design of the current study allowed data to be collected under standardized conditions while integrating the measurements into the everyday lives of the test subjects, resulting in an accurate representation of pH value. The pH probe used in the current study is an established, validated instrument for measuring pH in the esophagus during endoscopic examinations for reflux disease [22, 23]; pH measurement with this probe is thus standard, and it is a proven, safe, and reliable means of determining the pH value in the esophagus [24, 25]. The validation of the pH probe has also shown good reproducibility of the measurements in different pH solutions, and the results are in line with other data related to pH values in saliva and biofilm [16]. It shows, that in the established biofilm the pH values change in parallel with the saliva, but on a significantly lower pH-level, which may be expected due to the bacterial source of the acidic challenge. It was unexpected, though, that the relapse of the pH to neutral did not appear within 30 min, which would be expected in accordance with the Stephan-curve. One can speculate about the reasons for the deviations from Stephan’s curve. An interesting hypothesis could be the method chosen. The data was not collected under ‘laboratory conditions’ but in the everyday lives of the test subjects. It is also possible that the circadian rhythm (day/night) of the individuals and the existing oral microbiome had an influence. Similar results were observed in a study that investigated the relationship between pH value and caries risk in test subjects [26]. The results obtained when measuring pH value in an established biofilm (10 days) should be verified by further studies to better establish this new methodology [21]. Nevertheless, this new method for measuring pH in the mouth (wireless, 96 hours) could be integrated into the everyday lives of test subjects and thus reflect real-life conditions. This opens up exciting future fields of research, such as investigating the influence of the circadian rhythm and the female menstrual cycle on pH values in the oral cavity or investigating/monitoring diseases (e.g. reflux). Basically, our pilot study has shown that the chosen methodology could offer this possibility.

The current study has a number of limitations. Due to the exploratory study design and the small number of participants, the results are statistically significant but should be interpreted with caution. This means that further studies with a larger cohort should be conducted to test the procedure on heterogeneous groups of subjects (e.g. caries, salivation, reflux). However, this study was conducted as proof of principle, i.e. to test whether the chosen method works. Furthermore, due to the size and extent of the pH probes used, they were generally positioned in the posterior region, frequently in the lower jaw [27, 28] on the buccal side of the teeth and may not be valid for other areas in the mouth. However, the current study can be considered a model in which the feasibility of wireless 96-hour monitoring can be observed. Furthermore, we did not include the measurement of oral temperature with a temperature sensor in our study design. However, it is known that pH measurement depends to a certain extent on the ambient temperature [13, 14, 29]. To avoid this negative effect, the test subjects were not allowed to eat or drink anything for one hour before and after drinking the glucose. It can therefore be assumed that the mouth temperature remained constant during the measurement. Like all clinical investigations, this study relied on the cooperation of the test subjects. Thus, the results are only as good as the test individuals adherence to the study guidelines. Although detailed study information documents were used and close personal supervision was provided, it cannot be ruled out that the results may have been distorted by inaccuracies on the part of the test subjects when using the oral measuring devices. Although the study cohort was a mixed-gender group, it was relatively homogeneous in terms of other factors (age, plaque index). It would therefore be beneficial to test the probe in a larger cohort of subjects of patients with different risks of caries. In order to fulfil our duty of care towards the test subjects and comply with safety standards, the following continuity criteria were defined for the course of the study: a negative change in the DMFT value, PSI > 2. If either of these criteria was met, the participant would have had to withdraw from the study.

## Conclusions

pH measurements in saliva and biofilm are relevant for measuring cariogenic potential of foods. Until now, this has not been possible in vivo over a longer period of time in real-time scenarios. In this exploratory study, valid data were obtained over a period of 96 h. After consumption of a glucose solution, a significant difference was found in saliva and biofilm. Future studies should aim to include participants of different genders and from a wide age range with varying baseline oral health conditions in order to determine possible effects on pH levels.

## Electronic Supplementary Material

Below is the link to the electronic supplementary material.

Supplementary Material 1

## Data Availability

The datasets used and/or analyzed during the current study are available from the corresponding author (Antje Geiken, [geiken@konspar.uni-kiel.de] (mailto: geiken@konspar.uni-kiel.de) ) on reasonable request.
