# Supplementary material for: A new method for continuous in vivo pH measurement in saliva and oral biofilm - a comparative pilot study
Source: Clin Oral Investig. 2025 Dec 22;30(1):23. doi: 10.1007/s00784-025-06703-9 (PMC12719346; doi:10.1007/s00784-025-06703-9)
Supplement: Supplementary file 1 — Supplementary Material 1 [file 784_2025_6703_MOESM1_ESM.docx]

**Supplement**

Supplement Figure 1: Example of a test subject's pH levels over the course of a day (24h). blue: biofilm, orange: saliva. The green bar marks the times at which the glucose solution was taken. The pH value is shown on the x-axis and the corresponding time on the y-axis. note: The data collected during storage of the appliance in saline solution (food intake, oral hygiene) were not included in the evaluation.


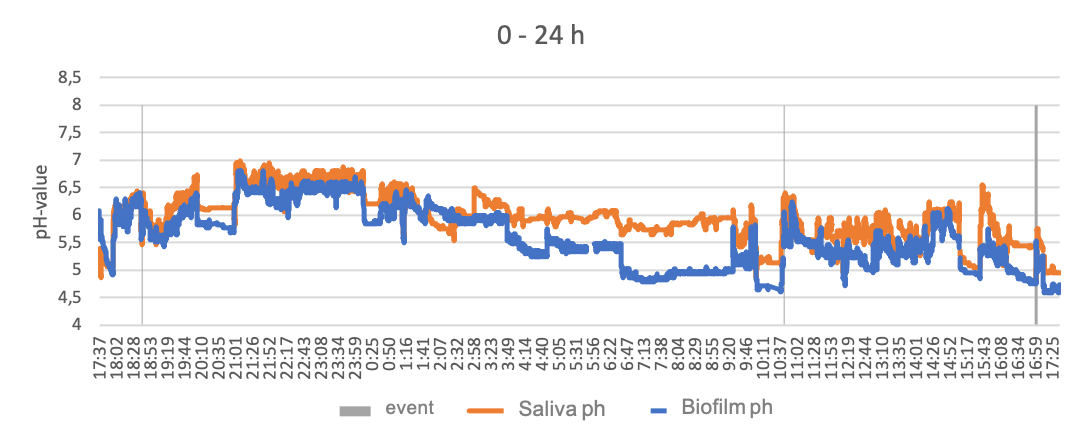


Supplement Figure 2: Example of the pH values of a test subject when drinking the glucose solution Blue: biofilm, orange: saliva. The pH value is shown on the x-axis and the corresponding time on the y-axis. The data collected during storage of the appliance in saline solution (food intake, oral hygiene) were not included in the evaluation.

Supplement Figure 3: Continous pH dynamics across all days and participants. Green: biofilm, orange: saliva. The pH value is shown on the x-axis and the corresponding time on the y-axis.
